# Wird der Einsatz der virtuellen Realität in der HNO-Heilkunde-Lehre von Studierenden automatisch positiv bewertet? Eine Fragebogenevaluation bei Studierenden

**DOI:** 10.1007/s00106-024-01453-8

**Published:** 2024-04-05

**Authors:** C. Offergeld, S. Kuhn, J. Kromeier, S. Heermann, A. Widder, O. Flayyih, F. Everad, A. Knopf, T. Albrecht, V. Burkhardt, T. Hildenbrand, W. Ramackers

**Affiliations:** 1 Univ.-HNO-Klinik, Medizinische Fakultät, Universitätsklinikum Freiburg, Freiburg, Deutschland; 2 Institut für Digitalisierung in der Medizin, Universitätsklinikum Gießen-Marburg, Marburg, Deutschland; 3 Klinik für Radiologie, St. Josef-Krankenhaus Freiburg, Freiburg, Deutschland; 4 Institut für Anatomie und Zellbiologie, Medizinische Fakultät, Universitätsklinikum Freiburg, Freiburg, Deutschland; 5 Studiendekanat der Med. Fak., Universitätsklinikum Freiburg, Freiburg, Deutschland; 6 Univ.-HNO-Klinik, Medizinische Fakultät, Universitätsklinikum Tübingen, Tübingen, Deutschland; 7 Klinik für Allgemein‑, Viszeral- und Transplantationschirurgie, Medizinische Hochschule Hannover (MHH), Carl-Neuberg-Straße 1, 30625 Hannover, Deutschland

**Keywords:** Medizinische Notfallversorgung, Simulationstraining, Regionale Anatomie, Fragebogen, Regressionsanalyse, Emergency medical services, Simulation training, Regional anatomy, Questionnaire, Regression analysis

## Abstract

**Hintergrund:**

Analog zu anderen Fachdisziplinen hat auch die digitale HNO-Heilkunde-Lehre deutliche Fortschritte während der Pandemiezeit verzeichnen können. So waren die HNO-Kliniken bundesweit vergleichsweise zeitig in der Lage, ein komplettes virtuelles Lehrangebot, auch unter Einsatz von innovativen Formaten, aufzubieten. Dies wurde entsprechend in studentischen Lehrevaluationen gewürdigt. Durch den zwischenzeitlich vermehrten Einsatz der virtuellen Realität (VR) in der Lehre sollte eine höhere Zufriedenheit durch eine Verbesserung der Lehrqualität zu erwarten sein.

**Material und Methoden:**

Es wurden Studierende (*n* = 180) nach Absolvierung des HNO-Blockpraktikums im Sommersemester 2023 mittels standardisiertem Fragenbogen befragt. Ziel der Evaluation war es, die Zufriedenheit und die Einschätzung der Effektivität der neu implementierten Methode VR zu ermitteln. Die VR wurde zur Vermittlung der Ohranatomie und der Koniotomie eingesetzt. Vergleichend wurde die VR mit den Inhalt Koniotomie auch bei Assistenzärzt:innen eingesetzt und evaluiert.

**Ergebnisse:**

Die Lehrveranstaltung wurde mit einem Mittelwert von 11,7 auf einer Punkteskala bis 15 (wie in der Oberstufe der Schulen) von den Studierenden positiv wahrgenommen. Die Effektivität und der Stellenwert des Einsatzes der VR im HNO-Lernportfolio wurden von den Studierenden unterschiedlich bewertet und fand nicht den erwarteten uneingeschränkten Zuspruch. Anders gestaltete sich die Beurteilung bei den Assistenzärzt:innen.

**Schlussfolgerung:**

Die VR stellt einen innovativen und vielseitigen Baustein im Lehrportfolio der HNO-Heilkunde dar. Diese neue Lehrmethode wird als zukunftsorientiertes Tool angesehen und akzeptiert. Dabei votierten die beteiligten Ärzte durchweg positiv, während die Studierenden im individuellen und subjektiven Bereich eher kritische Beurteilungen abgaben und Limitationen aufzeigten. Dies steht im Gegensatz zu den von den Interessenvertretungen der Studierenden geforderten Weiterentwicklung innovativer Lehrmethoden.

## Abstract (en)

**Background:**

Analogous to the situation in other disciplines, digital ENT teaching made significant progress during the pandemic. Most ENT clinics nationwide were able to offer a complete virtual teaching program in time. Innovative teaching methods were also used early on. This was recognized in student teaching evaluations. Due to the expansion of virtual reality (VR) in medical teaching, even greater satisfaction should be expected through improved teaching quality.

**Materials and methods:**

Surveys were performed with students (*n* = 180) of the ENT block internship in the summer semester of 2023. The aim of the evaluation was to determine the students’ satisfaction with and subjective effectiveness of the newly implemented VR digital teaching method for teaching ear anatomy and coniotomy. A survey was also carried out among resident physicians.

**Results:**

The ENT teaching was perceived favorably by the students, with an average rating of 11.7 out of 15. The learning effectiveness and the value of VR in the ENT learning portfolio was evaluated varyingly by the students. The physicians’ assessment was different, with a more positive perception.

**Conclusion:**

Virtual reality represents an innovative component in the teaching portfolio of otolaryngology. This new teaching method is viewed and accepted as a future-oriented tool. Remarkably, the physicians involved voted consistently positively, while the students gave more critical assessments and pointed out limitations in the individual and subjective areas. These findings are in contrast to the further development of innovative teaching methods demanded by student interest groups.

## Digitale Lehre

Die digitale Transformation betrifft die HNO-Lehre ebenso wie alle anderen Bereiche der medizinischen Ausbildung. Ein gewaltiger Sprung vorwärts wurde erzwungenermaßen durch die COVID-19-Pandemie ausgelöst. Mittlerweile ist dieser Zeitabschnitt Vergangenheit, und die präpandemischen Verhältnisse scheinen sich wieder einzustellen. Glücklicherweise scheinen aber die digitalen „Errungenschaften“ der Pandemiezeit zwischenzeitlich weitgehend fest implementiert und damit nicht mehr umkehrbar zu sein. Dementsprechend sind Weiterentwicklungen in der digitalen Lehre zu erwarten, die mit einer gestiegenen Zufriedenheit der Studierenden mit der Lehre einhergehen, da von den Studierendenvertretungen dieses auch gefordert wird [1]. Die HNO-Lehre in Freiburg hat im Bereich der virtuellen Realität (VR) bereits seit mehreren Jahren Erfahrungen gesammelt und die Methode der VR bereits seit nunmehr 5 Semestern im Studierendenunterricht routinemäßig eingesetzt [2, 3]. Hierbei gab es eine große Akzeptanz vonseiten der Studierenden [2, 3].

Bei der VR geht es aber nicht nur um den Einsatz dieser innovativen Methode, sondern um den medizindidaktischen Mehrwert im Vergleich zur konventionellen Vermittlung. Die VR war zum Zeitpunkt der Implementierung in der Lage, den Bereich der Mittel- und Innenohranatomie in plastischer, 3‑dimensionaler Form anschaulich und effektiv zu vermitteln [1]. Durch den Einsatz der VR wurde eine Verbesserung des Lernerfolgs bei Studierenden nachgewiesen [3], der auch bei HNO-Weiterbildungsassistenten im Rahmen einer multizentrischen Studie verifiziert werden konnte [4].

Die VR ist als Lehrmethode mit unterschiedlichen Zielsetzungen bei verschiedenen Zielgruppen variabel einsetzbar. Im Rahmen eines Trainings für HNO-Notfälle wurde die VR zur Simulation einer Koniotomie als Teil einer repetitiven Lernkonzeption zur Vermittlung festgelegter Handlungsabläufe eingesetzt [5]. Hierbei kam auch der Gamifizierungsansatz zum Tragen mit der Verwendung von in Computerspielen üblichen Elementen, wie das Sammeln von Punkten. Das Lernen soll durch Steigerung der Motivation und die virtuelle Lernumgebung positiv beeinflusst werden [5, 6].

Im Sommersemester 2023 wurden sowohl die überarbeiteten VR-Anwendungen „Otologie“ als auch „Koniotomie“ erstmals parallel in der gesamten Studierendenkohorte (*n* = 180) eingesetzt. Nach Absolvieren des HNO-Blockpraktikums erfolgte eine Gesamtevaluation aller teilnehmenden Studierenden mittels Fragebogen. Hieraus sollte der Gesamteindruck, aber auch die individuelle subjektive Einschätzung bezüglich der digitalen HNO-Lehre und der Wertigkeit der VR erhoben werden.

## Material und Methoden

Es wurden 168 von 180 Studierenden in die Evaluation eingeschlossen, die im Sommersemester 2023 das HNO-Blockpraktikum absolviert haben. Davon hatten 8 Studierende die Befragung abgelehnt, in 4 Fällen war der Fragebogen unvollständig ausgefüllt. Die Teilnahme an der Evaluation war ausschließlich freiwillig und ohne Benachteiligung im Fall einer Ablehnung. Zudem war der an die Studierenden verteilte Fragebogen vollständig anonymisiert ohne Möglichkeit der Rückverfolgung. Der ausgegebene Fragebogen enthielt 15 Items auf einer 7‑stufigen Likert-Skala sowie eine Gesamtbewertung mit 16 Stufen (0–15), den Noten der gymnasialen Oberstufe entsprechend. Ein positives Votum der lokalen Ethikkommission lag vor (EK UKF 23-1167-S1), wurde aber aufgrund der Erhebungsmodalitäten und Fragebogenkonstellation nicht benötigt.

Die Werte der Fragebogenitems wurden in ordinalen Werten erfasst. Eine deskriptive Beschreibung erfolgte über Mittelwert und Standardabweichung. Binäre Daten wurden über Gesamtzahl am jeweiligen Kollektiv abgebildet. Bei fehlenden Werten wurde für Variablen die jeweils schlechteste Bewertung angenommen und mit dem Zahlenwert 7 imputiert. Die dichotome Variable „Negativwahrnehmung“ wurde durch die schlechtesten 15 % der Bewertungen nach Gesamtnote definiert. Zur Ermittlung der Einflussgrößen auf den dichotomen Endpunkt „Negativwahrnehmung“ wurde zuerst univariable und in der Folge dann multivariable binäre logistische Regression angewendet. Sämtliche Daten wurden mit der Statistiksoftware SPSS analysiert und ausgewertet.

## Ergebnisse

Es wurden 168 von 180 Fragebögen ausgewertet. Die Ergebnisse der deskriptiven Statistik sind in Tab. 1 dargestellt. Die binär-logistische Regression mit dem Endpunkt „Negativwahrnehmung“ (≤ 10 Punkte) konnte als Einflussfaktoren „Möglichkeit der individuellen Skalierung“, „Interesse an der HNO-Heilkunde durch digitale Lehre“ und „Beitrag der VR zum Lernerfolg“ identifizieren.

**Table 1:** 

Fragebogenitem	Anzahl *n*	Minimum	Maximum	Mittelwert	Standardabweichung
Ich konnte mein theoretisches Wissen über die Koniotomie in der VR anwenden	168	0	7	3,87	2,923
Die VR hat geholfen, meine praktischen Fähigkeiten zu verbessern	168	0	7	3,68	2,624
Die Intensität einer Notfallsituation wurde durch den Einsatz der VR gut vermittelt	169	0	7	3,33	2,499
Beim Lernen mit der VR-Simulation habe ich die Dreidimensionalität als hilfreich empfunden	168	0	7	4,25	2,596
Beim Lernen mit VR habe ich die Möglichkeit der individuellen Skalierung und Beweglichkeit als hilfreich empfunden	169	0	7	3,91	2,505
Meine Erwartungen an die digitale Lehre wunden dank VR erfüllt	169	0	7	3,89	2,408
Durch die Einführung der digitalen Lehre wurde mein Interesse an der HNO-Heilkunde erweckt	169	0	7	4,43	1,748
Mein vorhandenes anatomisches Wissen konnte ich gut mit der VR verknüpfen	168	0	7	4,58	2,151
Die VR hat zum Lernerfolg beigetragen	167	0	7	4,40	2,323
Ich war auch ohne die VR-Erfahrung an der HNO-Heilkunde interessiert	169	0	7	4,38	1,829
Meine Kompetenz in der Ohranatomie schätze ich VOR dem Training	166	1	6	3,19	0,964
Meine Kompetenz in der Ohranatomie schätze ich NACH dem Training	166	1	6	2,54	1,036
Meine Kompetenz in der Durchführung einer Koniotomie schätze ich VOR dem Training	166	1	6	3,97	1,503
Meine Kompetenz in der Durchführung einer Koniotomie schätze ich NACH dem Training	165	1	6	2,64	1,330
Gesamtbewertung der digitalen HNO-Lehre. (Punktesystem Oberstufe)	166	1	15	11,70	3,042

Es sind für jedes Fragebogenitem Minimum, Maximum und die Mittelwerte mit Standardabweichung dargestellt

*VR *virtuelle Realität

Die deskriptiven Statistiken der Fragebogenitems bestätigen eine Tendenz zur Mitte bei Verwendung einer 7‑stufigen Likert-Skala (Tab. 1). So schwankte der Mittelwert der Fragen 1–7 zwischen 3,33 und 4,43. Auch die Fragebogenitems 8–10 wiesen Mittelwerte zwischen 4,38 und 4,58 auf. Bei den Fragebogenitems zur Selbsteinschätzung vor und nach spezifischer VR-Anwendung (Fragebogenitems 11–15) lagen die Mittelwerte im Post-Trainings-Bereich bei 2,54 und 2,64. Die Prä-Trainings-Werte hingegen lagen bei 3,19 respektive 3,97 (Tab. 1). Die Gesamtbewertung der digitalen HNO-Lehre wurde nach dem Punktesystem der Oberstufe (0–15) ausgerichtet. Der Mittelwert lag hier bei 11,70 (Tab. 1).

Die Verteilung der Gesamtbewertung erfolgte anhand eines Balkendiagramms (Abb. 6).

**Figure 1:**
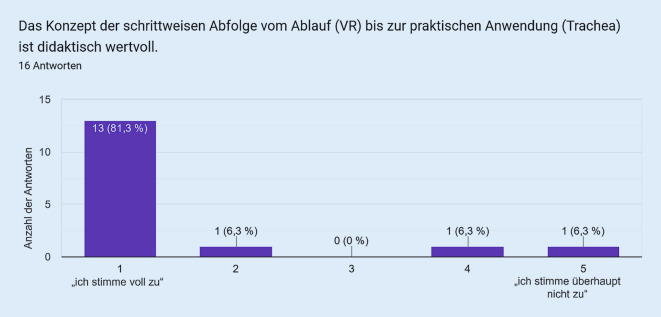

**Figure 2:**
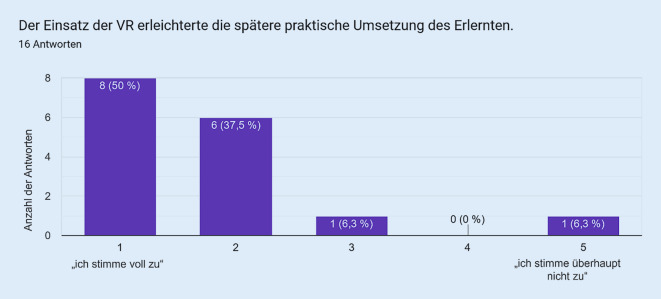

**Figure 3:**
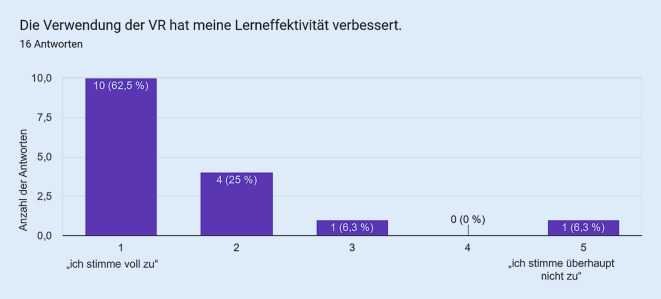

Als Vergleichsgruppe zu den Studierenden wurde die VR bei in der Notfallmedizin tätigen Assistenzärzt:innen eingesetzt. Die Fragebogenitems mit den korrespondierenden Antworten sind in den Abb. 1, 2, 3, 4 und 5 dargestellt. Hierbei zeigte sich eine positive Wahrnehmung der VR-Anwendung.

**Figure 4:**
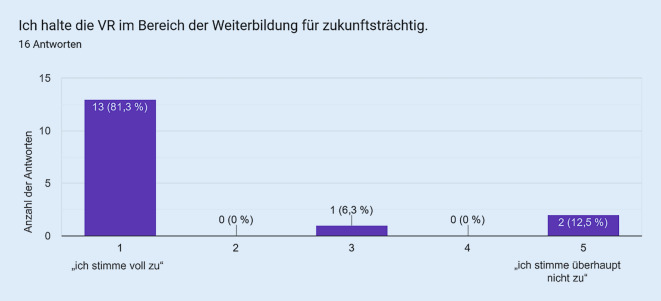

**Figure 5:**
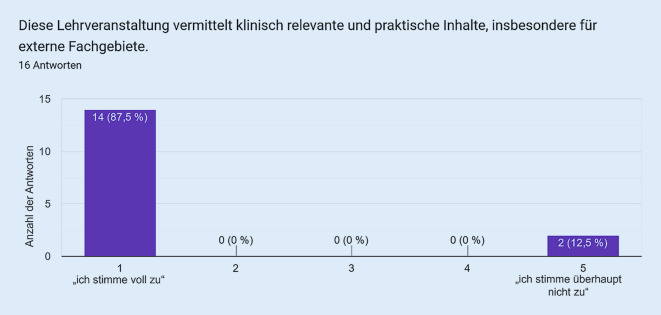

## Diskussion

In Zeiten knapper Ressourcen sind innovative Lehrkonzepte gefragt. Die Aus- und Weiterbildung in der HNO-Heilkunde ist ressourcenintensiv, obwohl es sich um ein kleines operatives Fach handelt. Das Fakten- und Handlungswissen muss aufgrund des breit gefächerten Fachgebiets in den Bereichen Ohr, Nase und Hals vermittelt werden. Dieses fängt schon bei den anatomischen Grundlagen an und erstreckt sich bis zur klinischen Anwendung in einer Notfallsituation wie der Koniotomie.

In diesem breitem Anforderungsspektrum kann die VR als Methode zur Vermittlung von Faktenwissen (z. B. Anatomie Ohr) und Handlungswissen (z. B. Koniotomie) sinnvoll eingesetzt werden. Der Einsatz der VR wurde von den Studierenden des Freiburger Curriculums positiv aufgenommen [2, 3]. Dieses ist gut nachvollziehbar, da die VR-Implementierung ein wichtiges Signal bei der offiziell vorgesehenen Umsetzung der digitalen Kompetenzen darstellt [7]. Diesbezüglich waren primär nur wenige Fachgebiete in der Lage, Erfahrungsberichte einzubringen [8, 9].

Der vielversprechende Ansatz mit der ersten Applikation „Otologie“ weckte große Erwartungen für diese innovative Lehrmethode [2, 3]. Auch bei der Anwendung der VR in einem Notfallszenario als Koniotomie gab es beträchtlichen Zuspruch, da hier Abläufe unter Zeitlimitierung in einem vergleichsweise realen Setting absolviert werden können [5].

Das VR-Modul Koniotomie wurde bei einer Workshop-basierten Fortbildungsveranstaltung für Notfallmedizin mit in der Notfallversorgung tätigen Ärzt:innen und Intensivpflegekräften eingesetzt, es wurde von den Teilnehmenden sehr positiv bewertet (Abb. 1, 2, 3, 4 und 5) und wies so den Weg zu weiteren Studien.

Bei der Analyse der deskriptiven Statistik der Ergebnisse der Studierendenevaluation vom Sommer 2023 fällt die Tendenz zur Mitte auf (Tab. 1). Dies kann der Tatsache geschuldet sein, dass eine 7‑stufige Likert-Skala verwendet wurde. Die Ergebnisse weisen aber auch darauf hin, dass sich die Einschätzungen der Studierenden von denen der Ärzt:innen unterscheiden. Entgegen der Annahme, dass der Einsatz neuer Verfahren von den Studierenden durchweg positiv bewertet wird, muss man verwundert auf die Ergebnisse schauen, da sie die stete Forderung der Interessenvertretungen der Studierenden [1] nach Implementierung innovativer Lehrmethoden relativieren.

**Figure 6:**
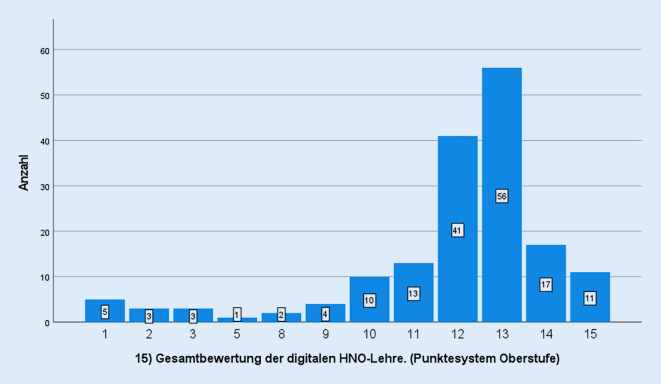

Die Fragen 1–10 hingegen belegen nicht nur die mittig orientierte Evaluation, sondern auch eine leichte Tendenz hin zur Ablehnung (Fragebogenitem 5). Das könnte darauf hinweisen, dass die Erwartungen der Studierenden nicht erfüllt wurden. Die mittig orientierte Evaluation der Fragebogenitems 1–10 weist vielmehr darauf hin, dass die digitale Lehre nicht um jeden Preis und schon gar nicht als Selbstläufer die Zufriedenheit der Studierenden steigert. Die positive Gesamtbewertung ist als Zustimmung zur HNO-ärztlichen curricularen Lehre zu werten, was aber nicht zwingend gleichbedeutend mit der digitalen Lehre sein muss (Abb. 6).

Der Einsatz der VR wird in der hier durchgeführten Evaluation von den Studierenden kritisch gesehen. Dies ist umso erstaunlicher, da in den Selbsteinschätzungen (Items 11–14) ein Trainingserfolg respektive Kompetenzerwerb wahrgenommen wird. Letztendlich bleibt festzuhalten, dass eine weitaus differenziertere Meinung zum Einsatz innovativer Lehrmethoden zu bestehen scheint. Die Studierenden scheinen auch trotz positiver Selbsterfahrungswerte dennoch eine kritische Haltung gegenüber VR einzunehmen. In diesem Zusammenhang wird auch ein Gamifizierungsansatz nicht unbedingt als positiver und/oder motivierender Lernaspekt angesehen, wenngleich mögliche positive Effekte auf das Lernverhalten bekannt sind [6].

Interessant ist bei dieser Erhebung die Erkenntnis, dass die Meinung der Studierenden durchaus stark variieren kann, wenn man erste Erhebungen mit den aktuell vorliegenden Daten vergleicht [3, 5]. Die alleinige Tatsache der Implementierung einer innovativen Lehrmethode reicht aktuell, im Vergleich zu 2020, nicht mehr aus, um ein durchweg positives Feedback von den Studierenden zu erlangen.

Ein mittlerweile ausgereiftes bzw. mehrfach überarbeitetes Lehrkonzept mit einem Fokus auf Faktenwissen, Handlungswissen und Kompetenzerwerb, welche von den Studierenden in den Umfragen innerhalb der letzten 3 Jahre als wichtig bzw. als zukunftsorientiert eingestuft wurden, kann hier nur eingeschränkt punkten.

Eine Kombination dieser Parameter wird nun im Wintersemester (WS) 2023/2024 von der universitären HNO-Klinik Freiburg pilotiert: Der erste Einsatz der VR zum Zweck der Vermittlung kommunikativer Kompetenzen. Auch dies war bereits Inhalt einer Stellungnahme der Interessenvertretung BVMD (Bundesvertretung der Medizinstudierenden in Deutschland e. V.) zur digitalen Lehre [1].

Übertragen auf die Weiterbildung stellen sich die Erfahrungen mit der VR bislang entschieden anders dar. Hier werden die neuen Lehrmethoden von den Weiterbildungsassistenten mit überaus großer Begeisterung und Motivation aufgenommen und – analog zu internationalen Studien – als immense Chance bzw. Bereicherung angesehen [10–14]. Dies kann darin begründet sein, dass die Weiterbildung – im Vergleich zur curricularen Lehre – über keine annähernd so strukturierte und operationalisierte Wissensvermittlung verfügt. Hier kann die oftmals nah an der Realität ausgerichtete VR als willkommene „Trainingsoption“ große Akzeptanz erreichen und wird dankbar angenommen.

Ein anderer Aspekt ist die unterschiedliche Situation von in der Notfallmedizin tätigen Ärzt:innen und Studierenden. Während bei den Ärzt:innen die Notwendigkeit des Erlernens des Ablaufs einer Notfallkoniotomie mittels VR auf der Hand liegt, ist das bei den Studierenden nicht so. Aufgrund der fehlenden Konfrontation mit der Situation in der Praxis wird das Erlernen als weniger relevant angesehen und somit in die Bewertung der Studierenden einfließen. In diesem Fall könnte der Gamifizierungsansatz sogar von Nachteil sein, da bei fehlendem Praxisbezug der spielerische Aspekt mehr in den Vordergrund rücken könnte.

Einschränkend muss man festhalten, dass die Anzahl der Studierenden (*n* = 168) die der Ärzt:innen (*n* = 16) deutlich übersteigt, sodass bei der Betrachtung der Ergebnisse diese berücksichtigt werden muss. Auch wenn die positive Beurteilung in der Gruppe der Ärzt:innen sehr eindeutig erscheint, sollte diese Anhand einer größeren Kohorte zukünftig verifiziert werden.

Die mittels binär-logistischer Regression ermittelten Einflussfaktoren für eine Negativwahrnehmung, „Interesse an der HNO-Heilkunde durch digitale Lehre“ und „Beitrag der VR zum Lernerfolg“ legen nahe, dass der Einsatz von VR in der HNO-Lehre nicht automatisch das Interesse am Fach steigert oder den Lernerfolg verbessert. Der Aspekt „Möglichkeit der individuellen Skalierung“ hängt mit der Zielgruppenorientierung des eingesetzten VR-Szenarios zusammen.

Hieraus lässt sich ableiten, dass ein kritisch hinterfragter, differenzierter und zielgruppenadaptierter Einsatz der VR für eine positive Wahrnehmung notwendig ist.

Es bleibt abzuwarten, ob diese Einschätzung sowie der mehrheitlich zu verzeichnende „Motivationsboost“ bei Weiterbildungsassistent:innen über einen vergleichbar schmalen Zeitkorridor ebenso einen reziproken Verlauf nehmen wird wie scheinbar in der Ausbildung von Studierenden. Da in der Weiterbildung eine wirkliche Strukturierung aber eher Seltenheitswert besitzt und der Lehre mehrheitlich kaum der angemessene Stellenwert eingeräumt wird, ist in absehbarer Zeit eher nicht davon auszugehen [15].
